# Neonatal events, such as androgenization and postnatal overfeeding, modify the response to ghrelin

**DOI:** 10.1038/srep04855

**Published:** 2014-05-06

**Authors:** Marta G. Novelle, María J. Vázquez, Kátia D. Martinello, Miguel A. Sanchez-Garrido, Manuel Tena-Sempere, Carlos Diéguez

**Affiliations:** 1 Department of Physiology, CIMUS, University of Santiago de Compostela-Instituto de Investigación Sanitaria (IDIS), 15782 Santiago de Compostela, Spain; 2 CIBER Fisiopatologia de la Obesidad y Nutricion (CIBERobn), Instituto de Salud Carlos III, Santiago de Compostela, Spain; 3 Department of Cell Biology, Physiology and Immunology, School of Medicine, University of Córdoba - Instituto Maimónides de Investigación Biomédica (IMIBIC)/Hospital Universitario Reina Sofia, Córdoba, Spain

## Abstract

It is currently accepted that ambient, non-genetic factors influence perinatal development and evoke structural and functional changes that may persist throughout life. Overfeeding and androgenization after birth are two of these key factors that could result in “metabolic imprinting” of neuronal circuits early in life and, thereby, increase the body weight homeostatic “set point”, stimulate appetite, and result in obesity. Our aim was to determine the influence of these obesogenic factors on the response to ghrelin. We observed the expected orexigenic effect of ghrelin regardless of the nutritional or hormonal manipulations to which the animals were subjected to at early postnatal development and this effect remained intact at later stages of development. In fact, ghrelin responses increased significantly when the animals were subjected to one of the two manipulations, but not when both were combined. An increased response to ghrelin could explain the obese phenotype displayed by individuals with modified perinatal environment.

GHRELIN, secreted basically in the stomach, is the most important orexigenic hormone described to date^1^,^2^,^3^,^4^. Both central and peripheral administration of ghrelin increases food intake significantly^5^. This effect is mainly mediated by an increase in NPY and AgRP expression and a decrease in POMC and CART. This physiological fact was established through elegant experiments in knockout (KO) mice. So, while NPY KO or AgRP KO showed a normal response in terms of food intake to ghrelin, the double KO, NPY/AgRP failed to show any response, indicating the existence of redundancy among these two neuropeptides as mediators of ghrelin orexigenic action^6^. The increase in NPY and AgRP requires activation of different transcription factors such as BSX (for both of them) and the forkhead box O1 (FoxO1) for *Agrp* gene and the phosphorylated cAMP response-element binding protein (pCREB) for *Npy* gene. In addition, data gleaned recently have uncovered a complex signaling pathway mediating ghrelin effects at hypothalamic level. These includes the energy sensors AMPK and SIRT-1, whose activation is dependent on p53^2^,^4^,^7^. In addition to its orexigenic effect at short term, ghrelin promotes adiposity^8^ and decreases locomotor activity^9^, lipolysis^10^, energy expenditure and activity of the sympathetic nervous system^8^,^11^. Moreover, ghrelin increases the preference for high fat diet^12^, stimulates the use of carbohydrates as an energy source and reduces the use of fat, promoting adipogenesis directly^8^,^13^,^14^. All these effects on adipose tissue metabolism are specific and independent of food intake and of growth hormone action^15^.

Due to the potential to block this potent orexigenic pathway, antagonists of ghrelin action have been proposed as anti-obesity therapies.

In addition to regulating energy homeostasis, ghrelin is a pleiotropic hormone that also has important effects during perinatal life on the development of the brain and its functional organization. Basically, it has a great effect on the neural and endocrine systems involved in metabolic regulation^16^,^17^. During the weaning period in rodents, circulating concentrations of ghrelin and leptin increase up to puberty (around 4 weeks of age). Both leptin and ghrelin can modulate NPY neuronal activity and the synaptic inputs into these neurons in an opposing manner^18^. Wortley et al.^19^ and Zigman et al.^20^ studies demonstrated, respectively, that deleting ghrelin and its receptor protects against diet-induced obesity in mice started on a high-fat diet in puberty, while adult mice are sensitive to obesity induced by diet. These results suggest important compensatory mechanisms and provide a possible potential role of the ghrelin system in hypothalamic development. Moreover, *in vitro* studies showed that ghrelin facilities neurogenesis in the spinal cord or hypothalamus cells only in the period immediately prior to, and after birth, but not in adult cells^21^. So, the exposure to factors that can alter ghrelin impact on development may induce lasting effects on its physiological regulation.

During perinatal life, pups are exposed to many environmental factors that can “programme” the energy regulatory system and have deleterious effects, such as hyperphagia, obesity and/or insulin resistance, among others^22^,^23^,^24^. In this regard, there are several studies showing that transient exposure to androgens during late gestation^25^ or an injection of exogenous testosterone after birth^26^,^27^,^28^ in female rats or female mice^29^,^30^ can cause metabolic imprinting. Furthermore, evidence suggests that a maternal androgen excess during pregnancy programs to develop polycystic ovarian syndrome (PCOS); the most common endocrine disorder in premenopausal women. So, neoanatal androgenization determines an increase in body weight and adiposity, insulin levels, triglycerides and cholesterol, as well as development of liver steatosis. This cardiometabolic dysfunction is due in part to excess of sympathetic tone^31^ and a state of systemic oxidative stress^32^. Therefore, it tenable that testosterone (either endogenous or exogenous), acting during early stages, has irreversible effects on the hypothalamus and, ultimately, on the regulation of food intake and energy expenditure^27^,^28^. Nohara et al, have demonstrated that exposure to testosterone in early life programs the hypothalamic melanocortin system. They observed that neonatal testosterone provokes a male phenotype of POMC neuron architecture and function that may cause an increase in female animals' food intake both in feeding as fasting conditions^29^. In addition, androgenized females present an impaired response to leptin in brown adipose tissue which may favour obesity development^31^. Hypothalamic KiSS1 neurons are also crucial targets and transmitters of the regulatory actions of sex steroids and metabolic cues during early organizing periods and adulthood^33^. In this context, it has been reported that perinatal testosterone excess modifies kisspeptin response impairing puberty and energy homeostasis both in female mice^29^ as in male mice^31^. By other hand, a series of studies also demonstrated that the amount of food consumed during suckling in the rat plays an important role in determining subsequent food intake in later life^22^,^34^,^35^. So, postnatal overfeeding might also modify the response to ghrelin after weaning.

In the above context, this study aimed to investigate whether neonatal androgenization, postnatal overfeeding or the sum of both manipulations in a female rats model, have effects on the orexigenic response to ghrelin in early stages of postnatal life and adulthood.

## Results

### Neonatal androgenization had orexigenic effect at early age and enhanced ghrelin orexigenic actions preferentially in adulthood

**Figure 1:**
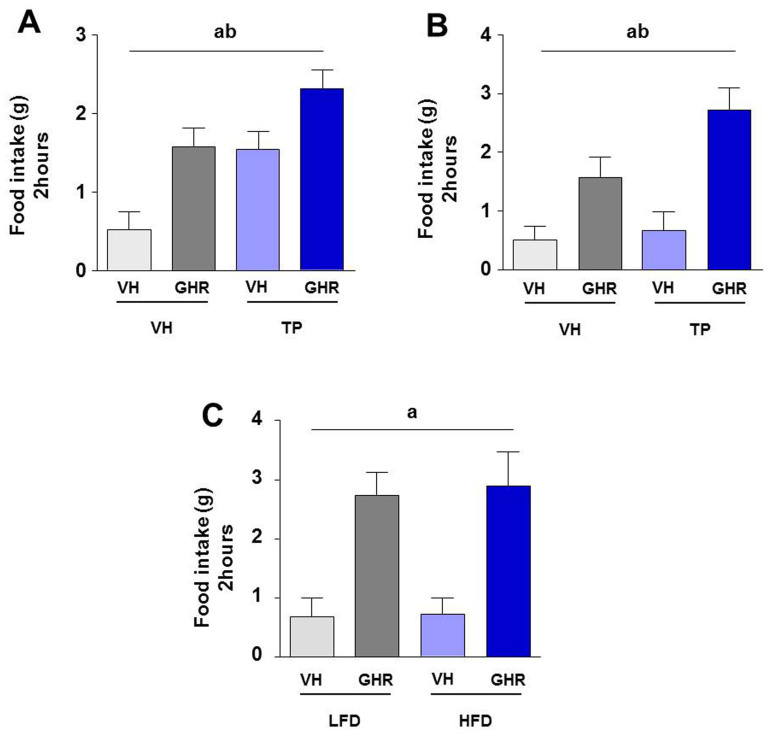
Cumulative food intake during 2 hours after intracerebroventricular (ICV) injection of 5 µl of saline (vehicle; VH) or 5 µl (1.5 nM) of ghrelin (GHR) in no androgenized (VH) and androgenized (testosterone propionate; TP) animals at postnatal day 24 (PND 24) (A) and postnatal day 90 (PND90) (B). Cumulative food intake during 2 hours after ICV injection of 5 µl of saline (vehicle; VH) or 5 µl (1.5 nM) of ghrelin (GHR) in androgenized animals subjected to LFD or HFD since PND24 until PND90 (C). Values represent the mean ± SEM. n = 7–12 animals/experimental group. Annotation indicates significant effect of a = ghrelin, b = neonatal androgenization.

Ghrelin-response studies were carried out in newly-weaned animals, postnatal day (PND) 24 (Fig 1A), and in adult animals, PND 90 (Fig 1B). As it was expected, according to data published by our group^2^,^4^,^7^, animals treated with ghrelin increased food intake significantly, both at PND 24 and 90, regardless of whether they had been androgenized or not. Notably, androgenized animals at PND 24 displayed basal levels of food intake that were similar to those of non-androgenized animals treated with ghrelin (Fig. 1A). We also obtained a significant effect due to neonatal androgenization (Two-way ANOVA), which in addition also exhibited in PND 90 (Fig. 1B).

### Ghrelin orexigenic effect is not modified in androgenized animals by high fat diet

Androgenized animals showed the same response to ghrelin regardless of whether they were fed on low-fat (LFD; 10% of calories from fat) or high-fat diet (HFD; 45% of calories from fat) from PND24 until PND90. Thus the response to ghrelin was assessed after 66 days exposure to HFD (Fig. 1C). This experiment confirmed that animals treated with ghrelin show a significant increase in food intake 2-h after administration of the hormone.

### Postnatal overfeeding enhanced the orexigenic effect of ghrelin

**Figure 2:**
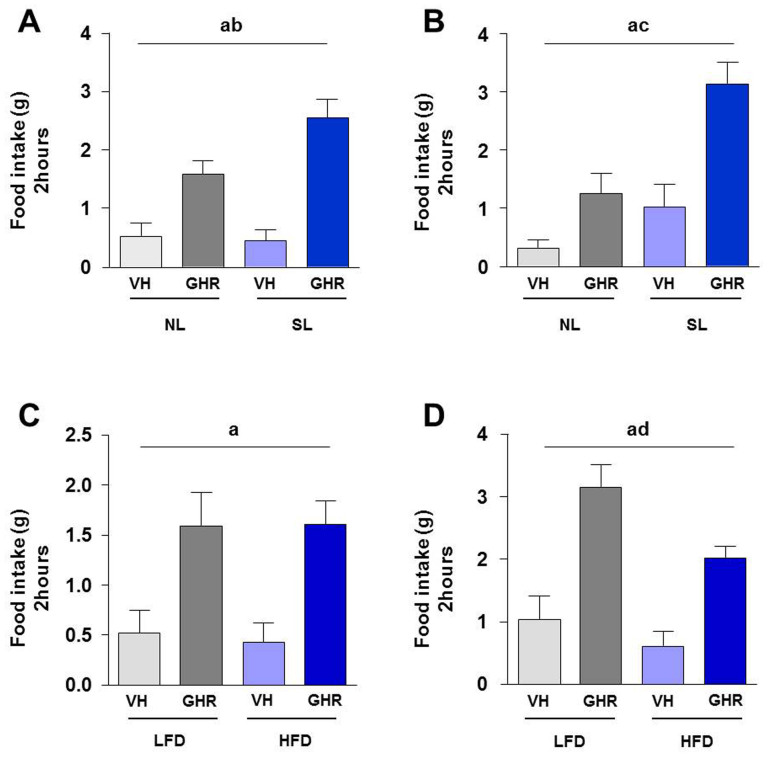
Cumulative food intake during 2 hours after intracerebroventricular (ICV) injection of 5 µl of saline (vehicle; VH) or 5 µl (1.5 nM) of ghrelin (GHR) in postnatal normofed (NL) and postnatal overfed (SL) animals at postnatal day 24 (PND 24) (A) and postnatal day 90 (PND 90) (B). Cumulative food intake during 2 hours after ICV injection of 5 µl of saline (vehicle; VH) or 5 µl (1.5 nM) of ghrelin (GHR) in normofed (C) and overfed (D) animals subjected to LFD or HFD since PND24 until PND90. Values represent the mean ± SEM. n = 7–12 animals/experimental group. Annotation indicates significant effect of a = ghrelin, b = interaction ghrelin-postnatal overfeeding, c = postnatal overfeeding, d = diet.

We observed that the response to ghrelin in animals subjected to postnatal overfeeding was significantly higher than in animals subjected to normal feeding during lactation. At PND 24 we observed that while without ghrelin injection SL animals slighted ingested less food than NL animals, the orexigenic effect elicited by ghrelin was bigger (significant interaction, Two-way ANOVA) (Fig 2A). In adulthood (PND 90; Fig 2B), we observed an extremely significant effect of analyzed factors, postnatal overfeeding and ghrelin injection, with SL animals showing higher basal food intake and an enhanced response to ghrelin.

### High fat diet modified ghrelin orexigenic response in postnatal overfed animals

These studies were carried out in adult PND 90 animals that had also been fed on LFD or HFD since weaning (PND24). As in previous studies, animals which had received ghrelin injection had a significant increase of food intake, regardless of the pattern of postnatal feeding [normal feeding (Fig. 2C) or postnatal overfeeding (Fig. 2D)], and independently of the type of diet received (LFD or HFD). Nevertheless, we observed that in overfed animals, HFD decreased the orexigenic effect of ghrelin in a significant way (Fig. 2D).

### Neonatal androgenization and postnatal overfeeding have no additive effect on the response to ghrelin

**Figure 3:**
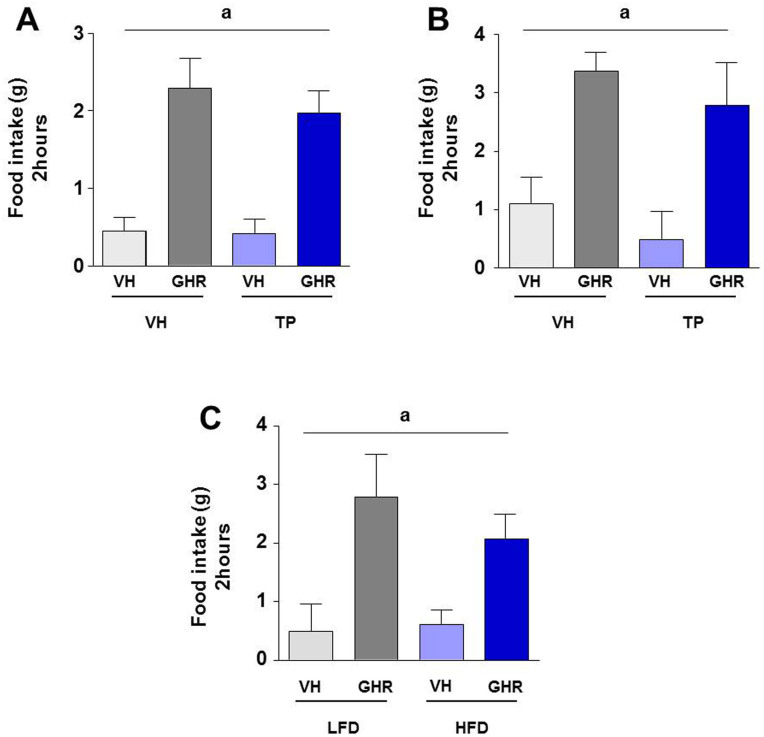
Cumulative food intake during 2 hours after intracerebroventricular (ICV) injection of 5 µl of saline (vehicle; VH) or 5 µl (1.5 nM) of ghrelin (GHR) in overfed animals during lactation and/or not androgenized (testosterone propionate; TP) at postnatal day 24 (PND 24) (A) and postnatal day 90 (PND 90) (B). Cumulative food intake in neonatal androgenized + postnatal overfed animals after injection of saline (VH) or ghrelin (GHR) in animals subjected to LFD or HFD since PND24 until PND90 (C). Values represent the mean ± SEM. n = 7–12 animals/experimental group. Annotation indicates significant effect of a = ghrelin.

When we analyzed the combined effect of both early hormonal (neonatal androgenization) and nutritional (overfeeding) manipulations, we did not detect a summative effect of these on the orexigenic effect of ghrelin. In fact, neonatally androgenized animals subjected to postnatal overnutrition displayed a trend for lower feeding responses to ghrelin than animals subjected to postnatal overfeeding alone; a trend that was detected at both PND 24 (Fig. 3A) and 90 (Fig. 3B), but without statistical significance When we analyzed the sum of another obesogenic factor, such as HFD, we only observed a significant effect of ghrelin (Fig. 3C).

## Discussion

Since its discovery, ghrelin has become an important focus of obesity research. It is known that it promotes food intake and a positive energy balance and facilitates the development of adiposity by decreasing fat oxidation^8^,^11^. The study of neuroendocrine network in the CNS involving ghrelin and its role during development may give us new points of view about its physiological function. In this study, we report that neonatal androgenization or postnatal overfeeding in a rat female model modify the orexigenic response to ghrelin both at early stages as in adulthood.

Notably, ghrelin ICV injection increased food intake significantly, regardless of the existence or not the other neonatal factors. Although there is no doubt of the role of estrogen in the regulation of energy balance, there are many studies that have examined the effects of ghrelin in females. In this context, studies carried out by Clegg's group documented that estradiol decreases the orexigenic ghrelin effect in female rats^36^, contrary to what was observed by our group^2^, and once again this study reaffirms. These discrepancies can be attributed to the different rat strain used (Long Evans *vs.* Sprague-Dawley) or the time of injection (1 h after starting the light phase *vs*. 6 h before starting the dark phase). In addition, although at low dose (0.01 nmol) they did not observe any orexigenic effect, when they increased dose (0.1 nmol or 1.0 nmol), they also observed ghrelin orexigenic effect in female rats.

Neonatal modifications, such as a transient exposure to high levels of androgen clearly impact on the organization of several metabolic and neuroendocrine functions^28^,^29^,^37^. So, when female rats were subjected to neonatal androgenization, these animals showed an increase in cumulative food intake after weaning. This effect might mediate in part by a masculinization of hypothalamic POMC neurons, since male exhibit a decreased density of POMC neuronal fibres and consequently a lower response to leptin^29^. This orexigenic effect of androgens was only observed at early stages, what might indicate that neuronal circuits are still establishing their “set-point”. Moreover, we observed that neonatal androgenization enhanced the ghrelin orexigenic response. In this sense, it has already been reported that male have a greater response to ghrelin ICV injection^36^.

We also found that postnatal overfeeding enhanced the orexigenic effect of ghrelin, both day 24 and 90. The development of hypothalamic circuits that regulate energy homeostasis takes place during lactation. Insulin and leptin act as trophic factors during this period, so it is believed that there is a causal relationship between hormone levels at this stage and altered persistent homeostatic system in adulthood. In this regard, it has been suggested that the hyperinsulinemia and hyperleptinemia in overfed animals program the hypothalamic circuitry, causing hyperphagia and increasing body weight^16^,^38^. Ghrelin itself has trophic effects during neonatal period^17^. Neonatal alterations could contribute to increase sensitivity to action of ghrelin, and consequently a condition of positive energy balance. Moreover, several studies have reported that overweight SL animals have changes in response to other circulating hormones. So, Davidowa *et al.* have demonstrated that postnatally overfed rats have altered responses to orexigenic and anorexigenic neuropeptides in paraventricular hypothalamic neurons^39^. By other hand, while under physiological conditions ghrelin inhibits expression of POMC through the action of GABA^40^, this mechanism may be altered in postnatal overfed rats since GABAergic circuits are modified^39^,^41^; a phenomenon that further documents the relevance of perinatal events to neuronal circuit development. In this sense, a change in sensitivity, expression or receptor location in GABAergic neurons might explain differences in the action of feeding-related peptides.

When we analyzed the effect of both neonatal factors, overnutrition and androgenization, we observed that there was not a cumulative increase in cumulative food intake. This result strongly suggests that, despite the individual impact of neonatal obesogenic manipulations, the established hypothalamic set point tends to keep food intake within a range that cannot be overcome by the summation of different obesogenic factors. The reasons for this are unclear at present. However it should be taken into consideration that each of these factors influence in different ways energy homeostasis. While overnutrition appears mainly to influence events mostly related to the so-called plasticity of hypothalamic neurons^18^, neonatal exposure likely involve more profound changes in neuronal development. Thus, rats overfed during perinatal life exhibited features of central leptin resistance as well as changes in different neuropeptidergic systems involved in the homeostatic regulation of energy homeostasis^34^,^35^. Neonatal exposure to androgens has been shown to exert marked alterations both at central and peripheral level in different signalling pathways involved in energy homeostasis. Neonatal androgenization in female have been shown to alters the development of central systems, neuropeptides and neurotransmitters, involved in the regulation of energy homeostasis, motivation and reward. In addition, it has been shown that they also led to long-lasting changes in serum leptin and corticosterone concentrations. Thus in the light of the marked changes induced by neonatal androgen exposure it is possible that the effects exerted by overfeeding are overcome. However due to the general lack of knowledge regarding on how these factors influence different transducing signals involved in food intake further work is needed before firm conclusions can be reached.

Data gleaned over the last few years have assessed the interaction between HFD and the ghrelin system. The importance of the ghrelin system in relation to HFD is highlighted by the finding that either ghrelin KO-19 or GHS-R –KO- mice^20^ are resistant to develop obesity. In addition, it have been reported that animals fed a HFD showed a decrease in total circulating ghrelin levels^42^,^43^,^44^ although they still showed a normal response in terms of fasting-induced increase in ghrelin levels^45^, indicating that the secretory capacity of ghrelin-producing cells is preserved. On the other hand, the impact of HFD in terms of the orexigenic effect exerted by ghrelin is more controversial. Data obtained in mice have reported certain degree of resistance to the orexigenic effect of ghrelin^46^,^47^,^48^. However, others studies carried out in rats failed to find out similar findings^45^. In addition, studies in human obese subjects showed a normal orexigenic effect of ghrelin despite overweight^49^. Whether these discrepancies are related to interspecies differences, differences in the types of HFD (40% to 60% HFD), length of exposure or the experimental model to hyperghrelinaemia (different doses, route of administration, chronic genetically-induced hperghrelinaemia, etc.) is yet to be clarified. The complexity of this issue is highlighted by our present data showing that ghrelin orexigenic effect can be influenced in different ways in relation to dietary and hormonal manipulation at early stages of development and in adulthood. Thus, while perinatal overfeeding increased the orexigenic effect of ghrelin in juvenile rats, it decreased this response in animals exposed to HFD during adulthood. Taken together, these findings and previously published data convincingly suggest that changes in the orexigenic actions of ghrelin are part of an adaptive response in order to face alterations in energy balance. This is in keeping with data showing a rapid rewiring in the hypothalamic mechanism influencing food intake, as shown by changes in the numbers of excitatory and inhibitory synapses and postsynaptic currents onto neuropeptide Y and POMC neurons following exposure to different nutritional and hormonal manipulations^18^.

In summary, our data showed that ghrelin-induced food intake is influence by factors such as neonatal androgenization, postnatal overfeeding and exposure to HFD during adulthood. Taken together, these findings may contribute to deepen in the knowledge of hypothalamic mechanism underlying to development of metabolic abnormalities that are linked to pathophysiological settings such as polycystic ovary syndrome and/or obesity.

## Methods

### Animals and experimental procedure

All experiments were carried out in accordance with the guidelines of the Spanish Committee for Experiments on Animals. All procedures performed were also approved by the University of Santiago de Compostela Institutional Bioethics Committee, the Xunta de Galicia (Local Government) and the Ministry of Economy and Competitiveness.

Female Sprague–Dawley rats were housed in a 12-h light: 12-h darkness cycle in a temperature- and humidity-controlled room. Chronic intracerebro-ventricular (ICV) cannulae were implanted in lateral ventricle with the animal under ketamine/xylazine anesthesia, as it was described previously^50^,^51^. After surgery, that took place at PND20 or PND85, the animals were placed directly in isolation test chambers for 4–5 days and were given free access to regular rat chow and tap water. Thereafter, the animals continued to have food available *ad libitum*. On the day of the experiment (PND24 or PND90), the animals received either an ICV administration of vehicle (5 µl of saline) or ghrelin (5 µg = 1.5 nmol; Bachem, Bubendorf, Switzerland) in a total volume of 5 µl. Animals were treated at 09:00 AM (one hour after the light cycle had commenced), when they were satiated. We measured food intake two hours later. We used 8–10 animals per group.

### Experimental design

#### Exp. 1. Effect of neonatal androgenization on responses to ghrelin

The model employed has been previously reported^28^,^52^. To induce neonatal androgenization female pup rats received subcutaneous injection of 1.25 mg testosterone propionate (TP) (T-1500, Sigma) diluted in 100 µl of olive oil on postnatal day (PND) 1. Studies of ghrelin response were done at PND 24, after weaning, or at PND 90. In order to analyze the combined effect of neonatal androgenization and high fat diet, since PND 24 until PND 90, the animals were fed with fat diet (HFD) (D12451, 45% calories from fat, 20% from protein and 35% carbohydrate; 4.73 kcal/g) or with low fat diet (LFD, control; D12450B, 10% of calories from fat, 20% from protein and 70% from carbohydrate; 3.85 kcal/g; Research Diets, Inc., New Brunswick, NJ).

#### Exp. 2. Effect of postnatal overfeeding on responses to ghrelin

The litter size was adjusted to induce early postnatal over- or normal feeding; being either, small litters (SL) with 3–4 rats in each litter (overfeeding) or normal litters (NL, control) with 12 rats (normal feeding), according to previously described procedures^34^,^35^. After weaning, the animals were fed with HFD or LFD until PND 90 to analyze the combined effect of postnatal overfeeding and high fat diet.

#### Exp. 3. Combined effect of neonatal androgenization and postnatal overfeeding on responses to ghrelin

In order to study the combined effect of both obesogenic factors a group of animals were subjected to neonatal androgenization followed by postnatal overfeeding, as we described above. We also studied the HFD effect on these animals. Body weight of the different experimental groups at PND 24 was as follows (mean ± SEM): VH-NL 55.825 ± 1.266 g; TP-NL 53.062 ± 0.849 g; VH-SL 70.797 ± 1.170 g; TP-SL 66.765 ± 1.041 g. Significant differences were observed between VH-NL *vs*. VH-SL p<0.0001; VH-SL *vs*. TP-SL p = 0.0109; TP-NL *vs.* TP-SL p<0.0001, VH-NL *vs.* TP-SL p<0.0001. At PND 90 body weight of the different experimental groups were as follows (mean ± SEM): VH-NL-LFD 274.900 ± 6.787 g; VH-NL-HFD 296.391 ± 8.172 g; TP-NL-LFD 302.042 ± 10.383 g; TP-NL-HFD 326.826 ± 13.090 g; VH-SL-LFD 298.750 ± 8.508 g; VH-SL-HFD 357.625 ± 8.993 g; TP-SL-LFD 305.000 ± 10.622 g; TP-SL-HFD 333.23 ± 11.623 g. Significant differences were observed between VH-NL-LFD *vs.* VH-NL-HFD p = 0.05; VH-SL-LFD *vs.* VH-SL-HFD p<0.0001; VH-NL-LFD *vs.* VH-SL-LFD p = 0.0391; VH-NL-HFD *vs.* VH-SL-HFD p<0.0001.

### Statistical analysis

All data were analyzed by two-way ANOVA test. Data are expressed as mean ± SEM and analyzed using *Graph Pad Prism* **5** for Windows (San Diego, California, USA).

## Author Contributions

M.G.N., M.T.S. and C.D. designed experiments. M.G.N., M.J.V., K.D.M. and M.A.S.G. performed experiments and analyzed the data. M.G.N. and C.D. wrote the paper. All contributors critically reviewed and approved the manuscript.
